# Increasing plasmid-based DNA vaccine construct (16 kb pSVK-HBVA) production in *Escherichia coli* XL10-Gold through optimization of media component

**DOI:** 10.1080/13102818.2014.989103

**Published:** 2015-01-22

**Authors:** Yu Wang, Liang Zhang, Wei Zhang, Hao Wu, Xiao Ming Zhu, Yuan Ji Xu, Jin Qi Yan, Ji Yun Yu

**Affiliations:** ^a^ Institute of Basic Medical Science, Academy of Military Medical Sciences, 27 Tai Ping Road, Beijing100850, China

**Keywords:** fermentation media, DNA vaccine, *Escherichia coli*, media component optimization

## Abstract

At present, there are production processes to produce protein by *Escherichia coli* (*E. coli*) fermentation. Research on the design and optimization of the plasmid fermentation medium, however, is less advanced. The fermentation medium that is optimized for plasmid DNA production is different from the medium that is optimized for protein production. So, establishing a scientific and rational method to optimize the fermentation medium used for plasmid production is very important. Previously, our laboratory developed a novel therapeutic DNA vaccine (named pSVK-HBVA) for hepatitis B based on the alphavirus replicon, and found that *E. coli* XL10-Gold was the optimal host strain for the production of plasmid pSVK-HBVA. The aim of this study was to establish a scientific and rational method to optimize the fermentation medium used for plasmid production, and investigate the effect of growth medium composition on the production of plasmid pSVK-HBVA harboured in *E. coli* XL10-Gold, as well as to optimize the medium composition.

The one-factor-at-a-time experiments demonstrated that Luria-Bertani (LB) was the optimal basic medium. The optimal carbon source and nitrogen source were glycerol and home-made proteose peptone, respectively. Based on the Plackett–Burman (PB) design, proteose peptone, glycerol and NH_4_Cl were identified as the significant variables, which were further optimized by the steepest ascent (descent) method and central composite design. Growth medium optimization in 500-mL shake flasks by response surface methodology resulted in a maximum volumetric yield of 13.61 mg/L, which was approximately 2.5 times higher than that obtained from the basic medium (LB).

## Abbreviations

E. coli: *Escherichia coli*
*LB*: Luria-Bertani
*PB*: Plackett–Burman design
*CCD*: central composite design
*RSM*: response surface methodology
*HIV*: Human immunodeficiency viruses
*HBV*: hepatitis B virus
*CMV*: cytomegalovirus
*M9*: minimal media
*TB*: Terrific Broth

## Introduction

Plasmid DNA (pDNA) based vaccines have distinct advantages over live viruses and viral-induced vaccines. These advantages include high safety, low vector-induced immunogenicity and ease of production.[1] To date, veterinary DNA vaccines have been licensed for the treatment of West Nile virus in horses (West Nile Innovator),[2] infectious hematopoietic necrosis virus in salmon (Apex-IHN) [3] and melanoma in dogs (canine melanoma vaccine).[4] In addition, one gene therapy approach has been approved for reducing piglet mortality (growth hormone-releasing hormone therapy, LifeTide™SW5).[5] Many DNA vaccines, including ones targeting human immunodeficiency viruses, cancer and hepatitis B virus (HBV), have entered clinical trials.[6] The demand for pDNA has increased vastly in response to rapid advances in the use of pDNA in gene therapies and vaccines. Therefore, it is very important to develop a convenient, rapid, high-yield technology to manufacture pDNA.

In the laboratory, the production of pDNA generally begins with the design and construction of the appropriate pDNA vaccine expression vector and the choice of the host bacterium. Next, optimization is performed on the media components and fermentation conditions. In the final step, segregation and purification are performed.[6,7] Fermentation is a critical step in the process of pDNA production. The engineered bacteria carrying the plasmid must be grown at a high cell density to produce a high yield of product.[8] To achieve high cell density, the composition of the medium must be optimized and a special fermentation strategy (fed-batch fermentation) must be used.[9] Like chromosomal DNA, pDNA is also composed of a sugar-phosphate backbone and nitrogen-containing nucleotides (A, T, G, C). Unlike proteins, the main elements that compose DNA macromolecules are carbon, phosphorus and nitrogen.[10] According to the central dogma of molecular biology, DNA synthesis requires only replication; however, transcription and translation are more complex mechanisms involved in the process of protein synthesis. Thus, the fermentation medium that is optimized for pDNA production is different from the medium that is optimized for protein production.[11]

The considerations discussed above demonstrate that the design and screening of a suitable fermentation medium for an engineered strain of *Escherichia coli* (*E. coli*) is crucial for achieving improved production of plasmid. At present, there are large-scale production processes to produce protein by *E. coli* fermentation. Research on the design and optimization of the plasmid fermentation medium, however, is less advanced.[10] Establishing a scientific and rational method to optimize the fermentation medium used for plasmid production is very important. The response surface method (response surface methodology (RSM)) is a commonly used statistical technique for designing experiments, modelling and discovering the conditions that best produce a desired response factor.[12] Plackett–Burman (PB) design is usually used in the first step of optimization to identify which variables are significant. These variables are further optimized by the steepest ascent (descent) method and central composite design (CCD).[13]

Previously, our laboratory developed a novel therapeutic DNA vaccine (named pSVK-HBVA) for hepatitis B based on the alphavirus replicon, and found that *E. coli* XL10-Gold was the optimal host strain for the production of the replicon DNA vaccine plasmid.[14] The objective of this study was to investigate the effect of growth medium composition on the production of plasmid pSVK-HBVA harboured in *E. coli* XL10-Gold, as well as to optimize the medium composition. One-factor-at-a-time experiments demonstrated that LB was the optimal basic medium and that the optimal carbon source and nitrogen source were glycerol and home-made proteose peptone, respectively. Mineral salt and trace elements improved plasmid yield to some extent, but vitamin B1 had no significant effect. Based on the PB design, proteose peptone, glycerol and NH_4_Cl were selected as significant variables, which were further optimized by the steepest ascent (descent) method and CCD.

## Materials and methods

### Plasmids, bacterial strains and reagents

In this study, we used the 16 kb plasmid pSVK-HBVA, which contains a kanamycin-resistance marker and an HBV antigen fusion gene under the control of a eukaryotic cytomegalovirus promoter. The host strains *E. coli* XL10-Gold was purchased from Agilent Technologies, Beijing, China. The host strains were stored in 20% (v/v) glycerol at −80 °C. Tryptone and yeast extract were purchased from OXOID LTD. Proteose peptone, soy peptone and casein peptone were purchased from Beijing Shuangxuan Microbe Culture Medium Products Factory, Beijing, China. Other biochemical reagents were purchased from Sinopharm Chemical Reagent Co., Ltd, Beijing, China, and were of analytical grade.

### Growth media and culture conditions

LB medium (tryptone 10 g/L, yeast extract 5 g/L and NaCl 10 g/L), Terrific Broth (TB) medium (tryptone 12 g/L, yeast extract 24 g/L, and glycerol 0.4% in which 2.31 g/L KH_2_PO_4_ and 12.54 g/L K_2_HPO_4_ were added after autoclaving separately) and minimal media (M9)(NaCl 0.5 g/L, NH_4_Cl 1 g/L, KH_2_PO_4_ 3 g/L, 1 M CaCl_2_ 0.1 mL/L, 1 M MgSO_4_ 2 mL/L and 20% glucose or glycerol 10 mL/L) were prepared to use as basic cultivation media. Unless otherwise stated, all cultures were grown under selective conditions in the presence of kanamycin sulphate (30 mg/L). All media were sterilized by autoclaving for 20 min at 121 °C. The trace element solution consisted of FeCl_3_·6H_2_O (27 g/L), ZnCl_2_ (2 g/L), CoCl_2_·6H_2_O (2 g/L), Na_2_MoO_4_·2H_2_O (2 g/L), CaCl_2_ (0.76 g/L), CuCl_2_·2H_2_O (1.27 g/L) and H_3_BO_3_ (0.5 g/L) dissolved in 1.2 mol/L HCl.

For seed culture, the glycerol stock of the strain was inoculated into a test tube (30 mL) containing 5 mL of LB medium at a ratio of 1:100 and incubated for 12 h at 37 °C, 200 rpm. For fermentation, a 0.5% (v/v) inoculum was added to a 500 mL flask containing 100 mL of LB medium, TB medium or M9 medium. The culture was then incubated at 37 °C, 200 rpm for 15 h.

### Determination of dry biomass concentration and plasmid yield

The dry biomass concentration was determined using an analytical balance. Samples (5 mL) were collected from growing cultures and centrifuged at 12,000 rpm for 10 min. The pellets were washed twice with phosphate buffered saline (PBS), centrifuged and dried in an oven at 105 °C for 24 h.[14] Before analysing the mass, the pellets were equilibrated for a period in an exsiccator. Plasmid concentration was measured by UV spectrophotometry. Agarose gel electrophoresis was used to analyse the morphology of the plasmid and measure the proportion of supercoiled plasmid. All experimental data were based on the mean of three parallel samples.

### One-factor-at-a-time experiments

The purpose of these experiments was to screen carbon sources, nitrogen sources, inorganic salts and trace elements for use in the culture media of *E. coli* HBVA. First, LB, TB, M9 (glycerol) and M9 (glucose) were screened to identify the optimal basic medium for culturing engineered bacteria by flask fermentation. Next, the effect of different carbon sources, such as glucose, glycerol and mannitol, and different nitrogen sources, such as tryptone, soy peptone, casein peptone and proteose peptone, on the production of recombinant plasmid pSVK-HBVA from *E. coli* HBVA was compared. Finally, mineral salt, vitamin B1 and trace elements were added to the media to determine their effects on fermentation and plasmid yield.

### Screening for significant variables using Plackett–Burman (PB) design

PB experimental design was applied to screen for significant variables [15] that influence plasmid production. From the one-factor-at-a-time experiments, the nine media compositions were assigned as nine variables. To estimate the experimental error and check the adequacy of the first-order model, three insignificant dummy variables were added to the variables of real interest. Minitab software was used to design 21 group experiments of 12 factors and two levels to screen for significant variables. The high and low levels of each variable and the experimental design are shown in Tables 1 and 2, respectively. PB experimental design is based on the following equation [16]:

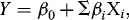

**Table 1.** Values of independent variables and the levels used in PB design.

			Levels		
Symbol	Independent variable	−1	0	+1	Unit
*X*_1_	Proteose peptone	5	10	15	g/L
*X*_2_	Yeast extract	2.5	5	7.5	g/L
*X*_3_	Dummy variable	−1	0	+1	
*X*_4_	NaCl	5	10	15	g/L
*X*_5_	Glycerol	0.25	0.5	0.75	g/L
*X*_6_	Dummy variable	−1	0	+1	
*X*_7_	Na_2_HPO_4_	6.4	12.8	19.2	g/L
*X*_8_	KH_2_PO_4_	1.5	3.0	4.5	g/L
*X*_9_	Dummy variable	−1	0	+1	
*X*_10_	Mg_2_SO_4_	0.12	0.24	0.36	g/L
*X*_11_	NH_4_Cl	0.5	1.0	1.5	g/L
*X*_12_	Trace elements	0.5	1.0	1.5	mL/L

**Table 2.** Plackett–Burman experimental design and the summary of plasmid DNA yield.

Run	*X*_1_	*X*_2_	*X*_3_	*X*_4_	*X*_5_	*X*_6_	*X*_7_	*X*_8_	*X*_9_	*X*_10_	*X*_11_	*X*_12_	Plasmid production *Y* (mg/L)
1	0	0	0	0	0	0	0	0	0	0	0	0	8.970
2	1	1	−1	1	1	−1	−1	−1	−1	1	−1	1	11.530
3	−1	1	−1	1	−1	1	1	1	1	−1	−1	1	2.067
4	1	−1	−1	1	1	−1	1	1	−1	−1	−1	−1	10.310
5	−1	−1	−1	1	−1	1	−1	1	1	1	1	−1	4.862
6	1	1	1	−1	−1	1	1	−1	1	1	−1	−1	6.215
7	−1	1	1	−1	−1	−1	−1	1	−1	1	−1	1	7.475
8	−1	−1	−1	−1	1	−1	1	−1	1	1	1	1	6.100
9	1	1	−1	−1	−1	−1	1	−1	1	−1	1	1	7.315
10	−1	−1	1	−1	1	−1	1	1	1	1	−1	−1	6.875
11	1	1	1	1	−1	−1	1	1	−1	1	1	−1	12.920
12	1	−1	1	−1	1	1	1	1	−1	−1	1	1	12.495
13	1	−1	−1	−1	−1	1	−1	1	−1	1	1	1	8.025
14	−1	−1	−1	−1	−1	−1	−1	−1	−1	−1	−1	−1	3.040
15	−1	1	−1	1	1	1	1	−1	−1	1	1	−1	9.510
16	1	1	−1	−1	1	1	−1	1	1	−1	−1	−1	8.535
17	−1	1	1	1	1	−1	−1	1	1	−1	1	1	10.490
18	−1	−1	1	1	−1	1	1	−1	−1	−1	−1	1	6.195
19	1	−1	1	1	1	1	−1	−1	1	1	−1	1	8.310
20	−1	1	1	−1	1	1	−1	−1	−1	−1	1	−1	10.050
21	1	−1	1	1	−1	−1	−1	−1	1	−1	1	−1	9.310

where *Y* is the predicted response value, β_0_ is the intercept of the model, β*_i_* is the linear coefficient and Χ_*i*_ is the independent variable. The plasmid volumetric yield of each group in the experiment was measured from three parallel samples. The average of the three samples was used as the response value.

A *t*-test of the experimental results was performed using Minitab software, which was also used to analyse the main effects of various factors and to generate a first-order regression equation. The variables that had a significant effect (*p* < 0.01) on plasmid production were subjected to further optimization.

### Path of steepest ascent (or descent)

The preliminary estimates of system operating conditions can be far from the actual optimum. In such cases, it is necessary to move quickly into the vicinity of the optimum by experimentation. The method of steepest ascent can be a good solution to this problem.[17,18] According to the first-order regression equation in PB design, the contours of the response surface are a series of parallel lines. The direction of steepest ascent is the direction in which the response, *Y*, increases the most rapidly. This direction is normal (perpendicular) to the fitted response surface contours.[19] Thus, the variable coefficient of the first-order regression equation determines the direction of the steepest ascent and the step width.[20] For example, if the coefficient is negative, then the level of the factor is decreasing. Larger coefficients correspond to smaller step widths, and vice versa. The highest point of the slope is used as the centre point for CCD.[21] The specific experimental design is shown in Table 3.

**Table 3.** Experimental design of steepest ascent and the summary of plasmid DNA production.

	Proteose peptone (*X*_1_) (g/L)	Glycerol (*X*_5_) (g/L)	NH_4_Cl (*X*_11_) (g/L)	Plasmid production (mg/L)
Base pointa^a^	5	0.25	0.5	
Step width	5	0.25	0.5	9.13
Experiment 1	5	0.25	0.5	11.70
Experiment 2	10	0.5	1.0	12.82
Experiment 3	15	0.75	2.0	11.02
Experiment 4	20	1.0	3.0	

^a^ −1 level in the PB design in Table 1.

### Optimization of significant variables using CCD

To find the optimal cultivation medium for plasmid production, CCD with five coded levels was used to locate the true optimal levels of proteose peptone, glycerol and NH_4_Cl. According to the results from PB experiment and steepest ascent experiment, the levels of the variables and the experimental design are shown in Table 4. The following second-order polynomial model was used to express the results of CCD [22]:

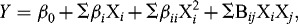

**Table 4.** Experiment design of CCD and the corresponding experiment data.

	*X*_1_ （proteose peptone）		*X*_2_ （glycerol）		*X*_3_ （NH_4_Cl）		
Run	Codes level (g/L)	Real level (g/L)	Codes level (g/L)	Real level (g/L)	Codes level (g/L)	Real level (g/L)	Plasmid production (mg/L)
1	−1	10	−1	0.5	1	3.0	12.06
2	0	15	0	0.75	1.68	3.68	11.73
3	0	15	−1.68	0.33	0	2.0	10.01
4	0	15	0	0.75	0	2.0	12.57
5	1	20	−1	0.75	−1	1.0	8.55
6	0	15	0	0.75	−1.68	0.32	7.63
7	1	20	−1	0.5	1	3.0	13.31
8	0	15	1.68	1.17	0	2.0	9.30
9	0	15	0	0.75	0	2.0	12.81
10	0	15	0	0.75	0	2.0	12.39
11	1	20	1	1.0	1	3.0	11.44
12	1.68	23.4	0	0.75	0	2.0	9.31
13	−1	10	−1	0.5	−1	1.0	8.00
14	−1	10	1	1.0	−1	1.0	7.00
15	−1	10	1	1.0	1	3.0	8.92
16	1	20	1	1.0	−1	1.0	9.41
17	−1.68	6.6	0	0.75	0	2.0	7.27

where *Y* is the predicted response value, and β_0_ *, *β*_i_*, β*_ii_* and β*_ij_* are the intercept of the model, the linear coefficient, the quadratic coefficient and the interactive coefficient, respectively. Χ_*i*_ and Χ_*j*_ are the coded independent variables.[23] Response surface regression was performed using Minitab software.

## Results and discussion

### Effects of different basic fermentation media on plasmid production and cell growth

To identify suitable basic fermentation media for the engineered bacteria, the engineered bacteria were grown in different media, including LB, TB, M9 (glucose) and M9 (glycerol). The plasmid concentrations and the OD value of the culture were measured. The highest plasmid yield, 5.5 mg/L (Table 5), was obtained using LB as the basic fermentation medium.

**Table 5.** Effects of different basic media on plasmid production and cell growth.

Medium	Dry biomass (g/L)	Volumetric yield (mg/L)	Specific yield (mg/g)
LB	0.63	5.5	8.7
TB	3.75	3.3	0.9
M9 (glycerol)	1.25	1.2	1.0
M9 (glucose)	0.21	0.5	2.4

**Figure 1.**
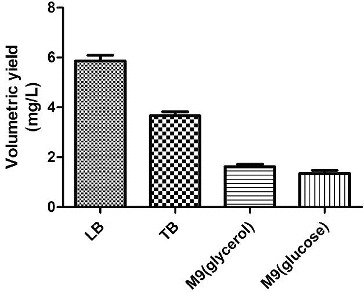
The volumetric yield of plasmid pSVK-HBVA obtained using different fermentation media.

Although using TB medium resulted in a higher bacterial biomass, the plasmid yield was less than that obtained using LB medium. The bacterial biomass and plasmid yield obtained using M9 medium were lower than those obtained using LB medium (Figure 1). Therefore, LB medium was selected as the basic fermentation medium in subsequent studies.

### Effects of different carbon sources on plasmid production

As a basic fermentation medium, LB contains no carbon source. The carbon source has a major impact on the growth of engineered bacteria and the plasmid yield. In this study, glycerol, glucose and mannitol were selected as candidate carbon sources, and their impact on the growth of engineered bacteria and on plasmid yield was studied.

**Figure 2.**
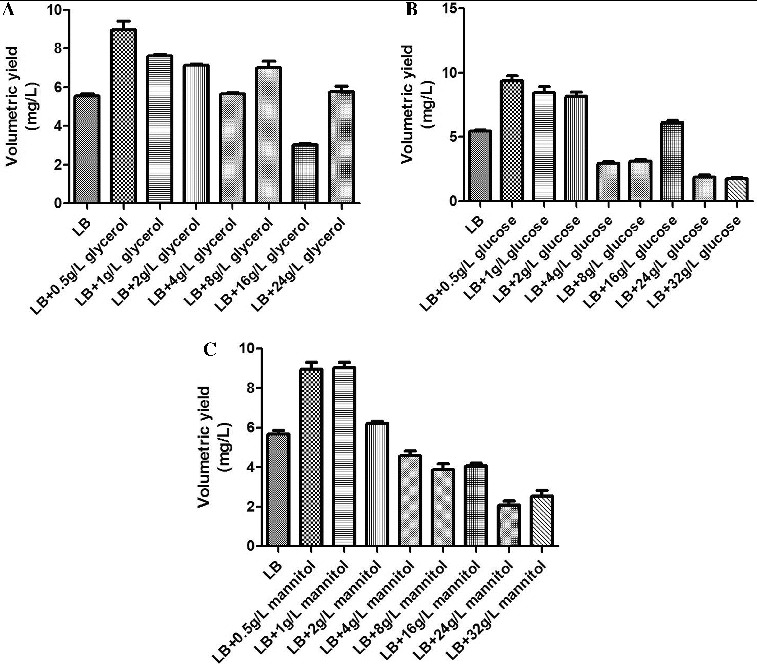
The effect of different carbon sources on the volumetric yield of plasmid pSVK-HBVA. (A) The effect of different concentrations of glycerol on the volumetric yield of pSVK-HBVA; (B) the effect of different concentrations of glucose on the volumetric yield of pSVK-HBVA; and (C): the effect of different concentrations of mannitol on the volumetric yield of pSVK-HBVA.

The experimental results demonstrated that a relative low concentration of carbon source could improve plasmid yield, but that an excessive concentration of a carbon source was not conducive to the production of plasmid. It is possible that in a high concentration of a carbon source, the engineered bacteria grew so rapidly that the rate of plasmid replication was less than the rate of bacterial growth, resulting in the loss of the plasmid. Glycerol, glucose and mannitol were the suitable carbon sources that produced similar results (Figure 2). Therefore, to save cost, glycerol was selected as the carbon source and was used at a working concentration of 0.5 g/L.

### Effects of different nitrogen sources on plasmid production

**Figure 3.**
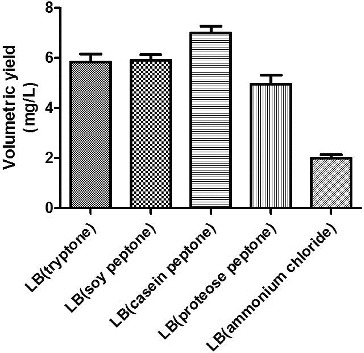
The effect of different nitrogen sources on the volumetric yield of plasmid pSVK-HBVA.

Tryptone, soy peptone, casein peptone, proteose peptone and NH_4_Cl were selected as candidate nitrogen sources, and their impact on the growth of engineered bacteria and on plasmid yield was studied. As shown in Figure 3, the highest plasmid yield was obtained using proteose peptone as the nitrogen source. Tryptone produced the next highest plasmid yield, and the minimum yield was obtained when inorganic nitrogen (NH_4_Cl) was used as the nitrogen source. Because tryptone was a costly imported product, domestically produced proteose peptone was selected as the nitrogen source for the fermentation medium. Proteose peptone was used at a working concentration of 10 g/L. The fermentation medium was called iLB.

### Effects of mineral salt, trace elements and thiamine hydrochloride on plasmid production

**Figure 4.**
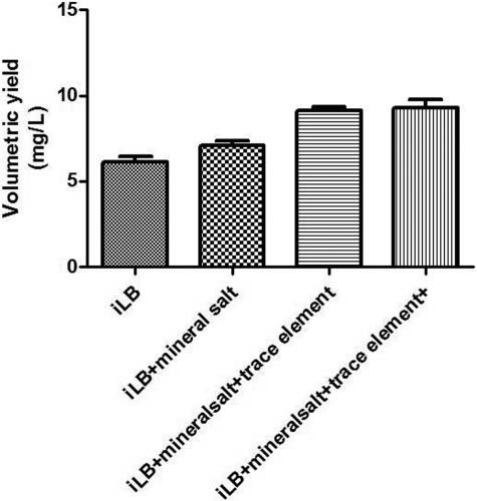
The effects of mineral salt, trace elements and thiamine hydrochloride on the volumetric yield of plasmid pSVK-HBVA.

In addition to carbon and nitrogen sources, engineered bacteria need inorganic salts, trace elements and vitamins to grow.[24] To investigate the effect of inorganic salts, trace elements and vitamins on the growth of the engineered bacteria and on plasmid production, inorganic salts, trace elements and vitamins were added into the fermentation medium and the resulting plasmid yields were measured (Figure 4). The addition of inorganic salts and trace elements improved the plasmid yield from the engineered bacteria, but the addition of vitamin had little effect on the plasmid yield.

### Selection of significant media components by process modelling using PB design

To identify the key factors that significantly affected the plasmid yield of the engineered bacteria, the nine variables (proteose peptone, yeast extract, sodium chloride, glycerol, Na_2_HPO_4_, KH_2_PO_4_, Mg_2_SO_4_, NH_4_Cl and trace elements) were investigated using PB design. These variables corresponded to columns *X*_1_, *X*_2_, *X*_4_, *X*_5_, *X*_7_, *X*_8_, *X*_10_, *X*_11_ and *X*_12_ in Table 1. Each factor contained a low level (−1) and a high level (+1). To examine the test error, another three virtual variables were designed. These variables corresponded to columns *X*_3_, *X*_6_ and *X*_9_ in Table 1. As seen from the results of the PB design (Table 6), the adjusted coefficient of determination, *R* ^2^, was 93.34%, indicating that the data variability was explained well by the models. The *p*-values of the models were less than 0.001, indicating that the models were significant.[25] In this study, a model term was considered significant when its *p*-value was less than 0.01. Proteose peptone, glycerol and NH_4_Cl were found to be significant model terms, indicating that these three variables were the variables of greatest importance for plasmid yield. Thus, proteose peptone, glycerol and NH_4_Cl were selected for further experimental study using the path of steepest ascent (or descent) method and CCD. By applying multiple regression analysis to the experimental data using Minitab software, the following first-order polynomial regression equation was established to explain the plasmid yield:

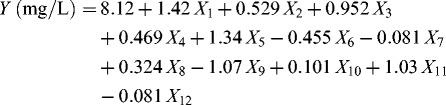

**Table 6.** Identification of significant variables for plasmid production using PB design.

Variables	Coefficient estimate	Standard error	*t*-value	*p*-value
Model		0.1629	24.33	0.000*
Intercept	8.081	0.1629	49.62	0.000
Proteose peptone	1.415	0.1629	8.69	0.000*
Yeast extract	0.529	0.1629	3.25	0.014
NaCl	0.469	0.1629	2.88	0.024
Glycerol	1.339	0.1629	8.22	0.000*
Na_2_HPO_4_	−0.081	0.1629	−0.50	0.633
KH_2_PO_4_	0.324	0.1629	1.99	0.087
Mg_2_SO_4_	0.101	0.1629	0.62	0.556
NH_4_Cl	1.026	0.1629	6.30	0.000*
Trace elements	−0.081	0.1629	−0.50	0.633

Note: *S* = 0.728326, PRESS = 31.1868, *R* ^2^ = 97.67%, *R* ^2^ (predicted) = 80.43%, *R* ^2^ (adjusted) = 93.34%.

*Model terms are significant.

where *Y* is the plasmid yield, *X*_1_ is the concentration of proteose peptone, *X*_2_ is the concentration of yeast extract, *X*_4_ is the concentration of sodium chloride, *X*_5_ is the concentration of glycerol, *X*_7_ is the concentration of Na_2_HPO_4_, *X*_8_ is the concentration of KH_2_PO_4_, *X*_10_ is the concentration of Mg_2_SO_4_, *X*_11_ is the concentration of NH_4_Cl, *X*_12_ is the concentration of trace elements and *X*_3_, *X*_6_ and *X*_9_ are the virtual variables.

### Path of steepest ascent (or descent)

The direction of steepest ascent and the step width were determined from the aforementioned first-order regression equation and the three important effect variables that were identified by PB design. Because the coefficients of these three significant variables were positive, the path of steepest ascent started from the lower level of the PB design and moved along the path in the direction in which the concentration of proteose peptone, glycerol and NH_4_Cl increased. The experimental results are shown in Table 3. The highest response value was achieved at the third step, when the proteose peptone concentration was 15 g/L, the glycerol concentration was 0.75 g/L and the NH_4_Cl concentration was 2.0 g/L. The high response value suggested that this point was near the region of maximum yield.

### CCD and response surface analysis

The effect of the levels and interactions of the significant media components (proteose peptone, glycerol and NH_4_Cl) on plasmid yield were further studied using CCD of the RSM. The experimental design of RSM (see Table 4) was performed using Minitab design software. From the experimental results (Table 4), multiple regression analysis of the experimental data (Tables 7 and 8) produced the following second-order polynomial equation:

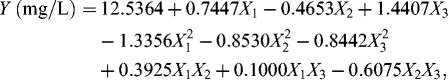



where *Y*_1_ represents the volumetric yield of the plasmid, *X*_1_ represents the concentration of proteose peptone, *X*_2_ represents the concentration of glycerol and *X*_3_ represents the concentration of NH_4_Cl. The model fit was measured by the coefficient of determination, *R* ^2^. The *R* ^2^ value was 94.90%, indicating that the model explained 94.90% of the variability in the response (Table 7). The statistical significance of the quadratic model was determined by the *F*-test of analysis of variance.[26] The model *F*-value was 14.47 and the *p*-value was less than 0.01, indicating that the model was significant. In addition, the lack-of-fit *F*-value was 15.92 and the lack-of-fit *p*-value was 0.06, indicating that the lack of fit was not significantly correlated with pure error (Table 8). These results suggested that the predicted value fit well with the experimental value, which indicated that this mathematical model was suitable for the simulation of plasmid production from engineered bacteria.

**Table 7.** Parameter estimates for CCD experiments.

Effect	Parameter estimate	Standard error	*t*-value	*p*-value
Intercept	12.5346	0.4145	30.243	0.000*
*X*_1_	0.7447	0.1947	3.826	0.006*
*X*_2_	−0.4653	0.1947	−2.390	0.048*
*X*_3_	1.4407	0.1947	7.401	0.000*
	−1.3356	0.2143	−6.234	0.000*
	−0.8530	0.2143	−3.981	0.005*
	−0.8442	0.2143	−3.940	0.006*
*X*_1_*X*_2_	0.3925	0.2543	1.543	0.167
*X*_1_*X*_3_	0.1000	0.2543	0.393	0.706
*X*_2_*X*_3_	−0.6075	0.2543	−2.389	0.048*

Note: *S* = 0.719381, PRESS = 27.0065, *R* ^2^ = 94.90%, *R* ^2^ (predicted) = 61.97%, *R* ^2^ (adjusted) = 88.34%.

*Model terms are significant.

**Table 8.** Analysis of variance for response surface quadratic model for plasmid production.

Source	Degree of freedom	Sum of squares	Mean square	*F*-value	*p*-value
Regression	9	67.3998	7.4889	14.47	0.001*
Linear	3	38.8772	12.9591	25.04	0.000*
Square	3	24.2578	8.0859	15.62	0.002*
Interaction	3	4.2649	1.4216	2.75	0.122
Residual	7	3.6226	0.5175	15.92	0.060
Lack-of-fit	5	3.5338	0.7068		
Pure error	2	0.0888	0.0444		
Total	16	71.0224			

*Model terms are significant.

To depict the impact of the three factors on the response value in a more intuitive manner, three-dimensional response surfaces and contour plots were generated (Figure 5). These plots illustrated the interactions between the two variables (the other variable was set to a level of 0). The shape of the contour plots (circular or elliptical) indicated whether the interaction between the two variables was significant or not.[27] Circular contour plots of response surfaces indicate that the interaction between the corresponding variables can be ignored, while elliptical or saddle-shaped contour plots suggest that the interaction between the corresponding variables is significant.[28–30] As can be deduced from the shape of the contour plot, the interaction between proteose peptone and NH_4_Cl had minimal significance. The lack of significance may be because these two components (an organic nitrogen source and an inorganic nitrogen source, respectively) were utilized in different ways during the process of plasmid production. The interaction between proteose peptone and glycerol was also not significant. In contrast, glycerol and NH_4_Cl had a significant interaction. Glycerol was used as the carbon source mainly to increase biomass, while NH_4_Cl was used as the inorganic nitrogen source to provide nitrogen for the nucleotides used in plasmid synthesis. Because such inorganic nitrogen sources may be utilized more fully and more quickly than other nitrogen sources, the plasmid yield changed as the growth rate of the engineered bacteria changed. Therefore, the presence of a significant interaction between glycerol and NH_4_Cl was possible. Figure 5. Three-dimensional plots and corresponding contour plots of the effect of three variables on plasmid pSVK-HBVA yield, when the effect of two variables was plotted, the other variable was set at the zero level. (A) Interaction of glycerol and ammonium; (B) interaction of proteose peptone and ammonium; and (C) interaction of proteose peptone and glycerol.

**Figure:**
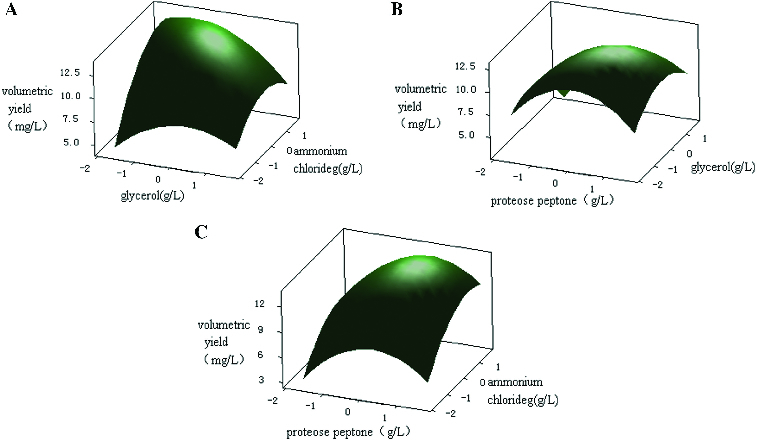

**Figure:**
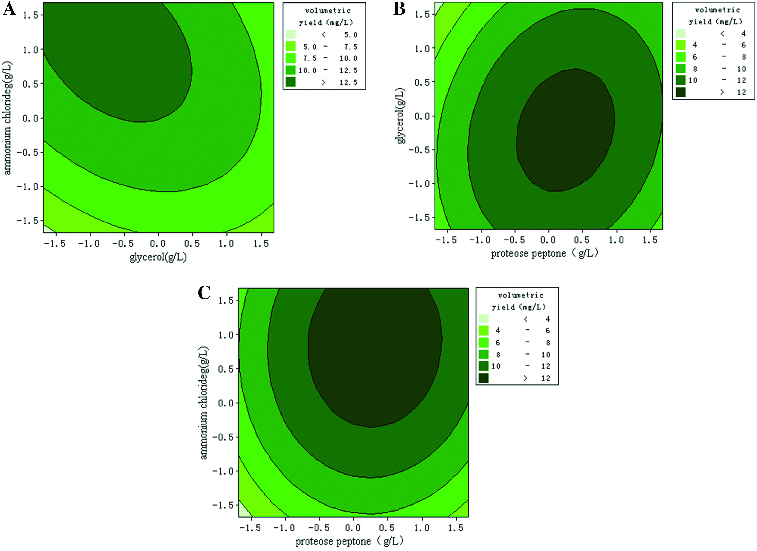


By calculating the partial derivative of the second-order polynomial equation using Minitab software, the optimal values of the three variables important for plasmid production were found to be 16.15 g/L for proteose peptone, 0.65 g/L for glycerol and 3.09 g/L for NH_4_Cl. Under the optimal condition, the predicted maximum plasmid yield was 13.54 mg/L. To confirm the degree of fit between the predicted value and the true value, three confirmation experiments were carried out using the optimal medium composition described above. The mean value of volumetric yield of plasmid was 13.18 ± 0.43 mg/L, which agreed well with the predicted value of 13.54 mg/L.

## Conclusion

Overall, production of pDNA is often hampered by the low plasmid volumetric yield which results from the unsuitable host bacterium or metabolic repressors. In this work, the bioprocess of medium optimization was employed, and successfully improved the production of pSVK-HBVA.

## Disclosure statement

No potential conflict of interest was reported by the authors.
